# Improved Progressive Polynomial Algorithm for Self-Adjustment and Optimal Response in Intelligent Sensors

**DOI:** 10.3390/s8117410

**Published:** 2008-11-19

**Authors:** José Rivera, Gilberto Herrera, Mario Chacón, Pedro Acosta, Mariano Carrillo

**Affiliations:** 1 División de Estudios de Posgrado e Investigación del Instituto Tecnológico de Chihuahua. Ave. Tecnológico No. 2909, Chihuahua Chih. México, 31310; Tel. +52 (614) 413 7474; Fax. +52 (614) 413 5187; E-Mails: mchacon@itchihuahua.edu.mx; pacosta@itchihuahua.edu.mx; mcarrillo@itchihuahua.edu.mx; 2 División de Estudios de Posgrado de la Facultad de Ingeniería de la Universidad Autónoma de Querétaro. Cerro de las Campanas S/N. Col. Las Campanas, Santiago de Querétaro Qro. México, 76010; Tel. +52 (442) 192 12 00 Fax. +52 (442) 192 12 00 ext. 6005; E-Mail: gherrera@uaq.mx

**Keywords:** Self-adjustment, calibration, interpolation, linearization, thermistor, smart sensor

## Abstract

The development of intelligent sensors involves the design of reconfigurable systems capable of working with different input sensors signals. Reconfigurable systems should expend the least possible amount of time readjusting. A self-adjustment algorithm for intelligent sensors should be able to fix major problems such as offset, variation of gain and lack of linearity with good accuracy. This paper shows the performance of a progressive polynomial algorithm utilizing different grades of relative nonlinearity of an output sensor signal. It also presents an improvement to this algorithm which obtains an optimal response with minimum nonlinearity error, based on the number and selection sequence of the readjust points. In order to verify the potential of this proposed criterion, a temperature measurement system was designed. The system is based on a thermistor which presents one of the worst nonlinearity behaviors. The application of the proposed improved method in this system showed that an adequate sequence of the adjustment points yields to the minimum nonlinearity error. In realistic applications, by knowing the grade of relative nonlinearity of a sensor, the number of readjustment points can be determined using the proposed method in order to obtain the desired nonlinearity error. This will impact on readjustment methodologies and their associated factors like time and cost.

## 1. Introduction

The development of intelligent sensors involves the use of self-adjustment algorithms that should be able to fix major problems such as offset, variation of gain and no linearity with good accuracy. Besides, it will be focused to simplify the calibration process.

The calibration stage is where many of the major problems begin because in the development and maintenance of measurement systems verification and readjustment processes are required. An accepted practice utilized in the past by measurement systems designers was to linearize the sensor output. The subject of linearization has been considered on different forms and stages, basically in the design of circuits with MOS and CMOS technologies [1-2]. Studied cases included the usage of analog to digital converters to solve nonlinearities at the same time that the conversion is made [3-4]. Also, type R-2R digital to analog converters have proven the necessity to improve the linear response [5]. Other research focused on improving the nonlinear response of specific sensors, like the thermistor [6] and the Hall Effect current sensor [7]. Further, ROM memories are used to save data tables and to solve the linearization problem [8-9]. Numerical methods have been developed using modern technologies [10-12]. The self-calibration concept using artificial neural networks is approached from different perspectives: for a pyroelectric sensor [13-14], simulation of autocalibration results [15] and works related with specific sensors autocalibration [16-18]. These can not be easily implemented on a digital signal processor (DSP) or a small microcontroller (μC). However, additional work on complex training process, a personal computer and special background preparation of the users are required.

Now, if the cost associated with the maintenance of the measurement systems is reviewed we can see that in a research done by Hutchins [19] at the end of the last century, he claimed that in the global marketing from the 70′s to the 90′s, the investment in test and measurement equipment reached 9,000 million dollars, from which, 2,000 million dollars were applied to maintenance and 1,200 million dollars were needed for the sole purpose of calibration. Companies' competitiveness must be certified. Initially, this was accomplished by the use of Military Standards and more recently with ISO's standards [20]. As a consequence, operational costs have increased. By the end of 2004, in manufacturing industries of the Unites States alone it was estimated that a total of 218.8 million dollars was spent on calibration services during 2005 [21] and by the year 2006 the budget on quality services with the sole purpose of anticipating problems and correcting measurement equipment was 1.1B dollars [22]. The characteristics of measurement systems can change according to different factors such as work environment, use, age, etc. In 2003, Powell [23] stated that “the calibration services industry has seen growth rates in excess of 15% per year in many countries”.

The development of intelligent sensors with readjustment capabilities is imperative in order to facilitate calibration while not increasing its costs. Today designers have different options for self readjustment sensors, some of which are artificial neural networks theory [16, 24] or recursive algorithms [25-26]

Several important works related to recursive algorithms that can be applied to the self readjustment of intelligent sensors exist, one of which is the progressive polynomial calibration method [25], although its effectiveness has not been proven yet and the number of points required to achieve a minimal error it is not clear. Another method used to select the number of readjustment points required is shown by Dias [26]. This particular calibration process is too expensive for the required number of points and the amount of time invested. Furthermore this criterion cannot be applied in a general way. Looking for a general solution that can be applied to different sensors, reducing the amount of time required for calibration process is a most. Further research is necessary to obtain an optimal result, and the minimum error with the lowest amount of verification points during readjustment process.

This paper presents the improvement of a progressive polynomial algorithm that facilitates the calibration process due to the self-adjustment of sensor. This proposal is based on the calibration method presented by Fouad [25]. The method has been improved in two aspects: the evaluation of the effectiveness method with respect to the percentage of nonlinearity of the input signal and the method's optimization to achieve the minimum error. In this proposal a minimal amount of adjustment points are taken into consideration and an evaluation of the correct selection sequence of the calibration points are made in order to get the optimal yield. To prove the worthiness of this proposal a real temperature measurement system was designed.

One important point that needs clarification before proceeding is related to the meaning of the term “self-adjustment”. Adjustment is mainly concerned to the process of removing systematic errors in accordance with the definition in the Metrology and the International Vocabulary of Basic and General Terms in Metrology (VIM), ISO VIM [27-28]. This action, in the past, was used as calibration by [4,15,24,25].

The paper structure will be the following: the basic system design considerations are presented in Section 2. The improved polynomial progressive algorithm and its simulation results are described in Section 3. A practical implementation of an intelligent sensor with improved algorithm on small microcontroller (MCU) is showed in Section 4 and the tests and results are described in Section 5.

## 2. Basics Considerations

### 2.1. Intelligent Sensors Functionalities

**Figure 1.**
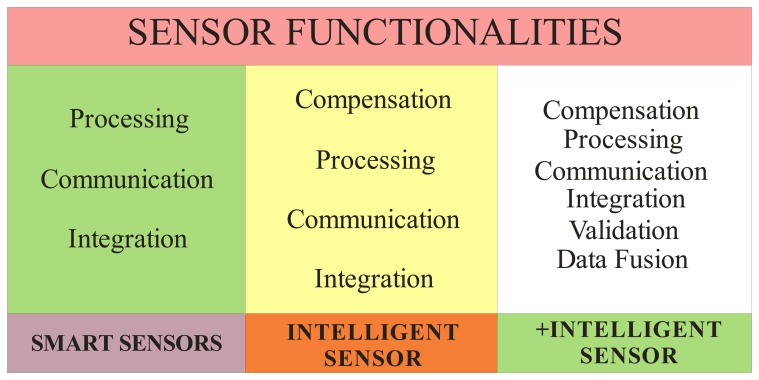
Intelligence Sensors classification base on its functionalities.

The term smart sensor was coined in the mid of 1980s [29] and sensor intelligence has been discussed since 1993 [30]. Using the references regarding intelligent sensors and the smart sensor definition from the Institute of Electrical and Electronics Engineers [29-30] a classification of intelligence in sensors based on their functionalities is proposed in Figure 1. Due to the importance of these aspects, they are considered individually, as an example, the cases of processing functionality [31-33].

This paper is focused on the compensation functionality. The compensation factor will be operating in three categories: Nonlinear compensation that linearizes the relationship between input and output, Cross-sensitivity compensation due to ambient conditions and time based or long term drift compensation due to degradation of the sensor or its elements [30]. An improved algorithm to make the compensation of the linearity and compensation due to degradation will be explained in the following sections.

### 2.2. Progressive Polynomial for Self-adjustment Method

A previous step to the application of progressive polynomial calibration algorithm is the normalization of the input and output variables. For example, the output electrical signal *y*′ of any sensor in response to the input variable *x*′ is defined by:

(1) y'=f(x')

In most of the cases the input and output variables scales are different, and these variables need to be normalized. We suggest making the normalization of *x*′ and *y*′ in the range of [0,1]. This can be obtained from the following equations:

(2) $$x=\frac{x'-x{'}_{\min}}{x{'}_{\max}-x{'}_{\min}}$$

(3) $$y=\frac{y'-y{'}_{\min}}{y{'}_{\max}-y{'}_{\min}}$$

After the normalization, Equation (1) can be rewritten as: *y* = *f*(*x*). Now, the desired output signal is one straight line with unitary slope. This will be the target in the adjustment process and will be the reference signal defined by:

(4) t=x

Once the input and output variables are normalized the four steps of the polynomial progressive polynomial method [25], described bellow, can be applied:

1. Based on the adjustment process of measuring systems [27], *N* readjustment points into of measurement range of the sensor are chosen and called readjustment vector, x′. The readjustment vector is supplied to the sensor and the output signal is recorded to generate the vector y′. Using Equations (2) and (3) the signals are normalized to get *x*(*i*) = [*x*_1,_*x*_2,…,_*x_N_*] and *y*(*i*) = [*y*_1,_*y*_2__,__…__,_*y_N_*] for *i* = 1 to *N*. The ideal output for each point is defined by Equation (4), *t*(*i*) = [*t*_1,_*t*_2__,__…,_*t_N_*]. These values are required only to compute the coefficients *k*_1_ to *k_N_* as described in the following steps.
2. With the first points *x_1_*, *y_1_* and *t_1_* any offset problem is fixed. This involves computing coefficient *k_1_*:
  (5) $$k_{1}=t_{1}-y_{1}$$
  and a new function *f_1_* is defined as follows:
  (6) $$f_{1}=y+k_{1}$$
  If the *y* function does not include an offset error, the coefficient *k_1_* is zero and the equation *f_1_* is equal to *y*. Remember, the normalized output signal for any input signal is represented by *y* and this signal is generated by the sensor under normal operation.
3. Using the points *x*_2_ and *y*_2_ the gain problem, if any, is eliminated. Where *x*_2_ is the upper limit of the measure scale. Then the *k_2_* coefficient is obtained by:
  (7) $$k_{2}=\frac{\left(t_{2}-f_{1}(x_{2})\right)}{f_{1}(x_{2})-t_{1}}$$
  The new function *f*_2_(*k*_2_,*t*_1_, *f*_1_), without the gain problem is defined as:
  (8) $$f_{2}=f_{1}+k_{2}(f_{1}-t_{1})$$
  If a gain error is not observed in *y*, the *k*_2_ coefficient is zero and equation *f_2_* is equal to equation *f_1_*.
  For sensors with linear transfer function three foregoing steps are enough to self-readjustment of the sensor, but if the transfer function is nonlinear, more coefficients *k* and functions *f* are required.
4. If the transfer function of the sensor is nonlinear, a new set of coefficients and functions are defined. The coefficients *k_3_* to *k_N_* are defined by:
  (9) $$\begin{array}{cc} k_{i}=\frac{\left(t_{i}-f_{i-1}(x_{i})\right)}{\prod_{j=1}^{i-1}\left(f_{j}(x_{i})-t_{j}\right)} & i=2,\ldots .,N \end{array}$$
  and the function *f_3_* to *f_N_* defined by:
  (10) $$\begin{array}{cc} f_{i}=f_{i-1}+k_{i}\prod_{j=1}^{i-1}\left(f_{j}-t_{j}\right) & i=2,\ldots .,N \end{array}$$
  The new transfer function of the sensor is represented by:
  (11) $$\left[\begin{array}{c} f_{3}\\ f_{4}\\ \ldots \ldots \\ f_{N} \end{array}\right]=\left[\begin{array}{c} f(k_{3},t_{1},\ldots ,t_{3},f_{1},f_{2})\\ f(k_{4},t_{1},\ldots ,t_{4},f_{1},\ldots ,f_{3})\\ \ldots \ldots \ldots \ldots \ldots \ldots .\\ f(k_{i},t_{1},\ldots ,t_{N},f_{1},\ldots ,f_{N-1}) \end{array}\right]$$

Finally, the relative error metric, (12), can be used to determine how linear is the *f_N_* function obtained:

(12) $$\varepsilon_{rN}=f_{N}-t(x_{\text{norm}})$$

Or another way to corroborate the method is by using the least mean square error, expressed by

(13) $$\varepsilon_{\text{mseN}}=\frac{1}{j}\sum_{i=1}^{j}{\left(t_{i}-f_{N}(i)\right)}^{2}$$

Current literature qualifies this method just as a qualitative way; the criteria of how the sequence of readjustment points will be taken and its capability of quantitative means are not available. In the next section one proposal for effective algorithm evaluation with respect to the percent of nonlinearity of the input signal is presented, as well as the necessary modifications to improve the method to obtain the minimum relative error.

## 3. Improved Progressive Polynomial Algorithm to Self-Adjustment

### 3.1. Permutation Vector Analysis

In the previous section a recursive linearization method was described. However, it is necessary to define its performance under a set of important characteristics. Some of these characteristics include: number of adjustment points required, time expended in the adjustment process, the minimum nonlinearity error desired, and the applicability of the method to any sensor.

For example, a criterion to choose the number of calibration points based on a scale region where the sensor will be used is described in [26]. This scale region is determined by analysis of the probability distribution function of the measurements. Nevertheless, for some measurement systems this method is expensive considering the calibration time and the number of required points. A new process to evaluate the algorithm's performance is described as follows.

**Figure 2.**
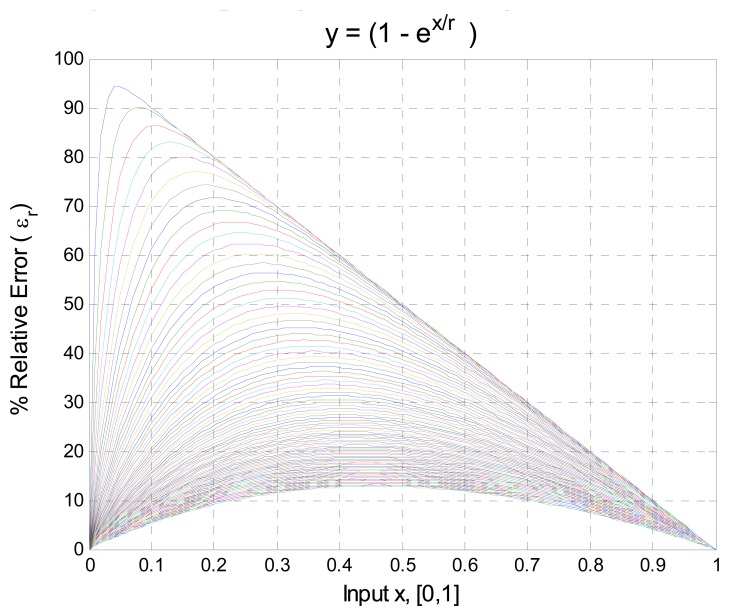
Input signal used for algorithm evaluation.

The first step is to select a nonlinear function as the input to the linearization process. In this document an increasing exponential function defined by (14) was selected:

(14) $$y'=(1-e^{-x'/r})$$

where *x*′ is the input signal of any variable, and the parameter *r* determines the increasing rate of the function. Therefore, *r* allows us to simulate different grades of relative nonlinearities. With the value of *r* the range of nonlinearity of the function *y*′ can be controlled and it may represent a great variety of sensors. Figure 2 illustrates several realizations of the Equation (14) showing different grades of relative nonlinearities, from 10% to 95% with *x*′ and y′ normalized according to Equations (2) and (3). The percentage of nonlinearity was computed with the Equation (12).

The next step consists of selecting a set of adjustment points, with cardinality *N*, for each calculation of (14). The order of the adjustment points is taken randomly or empirically, in this paper the number of selected points was *N*=*5* and *N*=*7*. For each set of adjustment points an input vector *x*(*i*) = [*x*_1_,*x*_2,__…__,_*x_N_*] is generated together with its corresponding *y*(*i*) = [*y*_1,_*y*_2,…__,_*y_N_*], and *t*(*i*) = [*t*_1,_*t*_2,…__,_*t_N_*] vectors. In order to reduce the computational burden and to standardize the process, *x*_1_ and *x*_2_ represent the lower and the upper limit that can be measured by the measurement system or the intelligent sensor.

Two sets of calibration points were randomly generated in order to consider two different cases. These sets are shown bellow:

**Case 1** with:
  *x_N_* = [0, 1.0, 0.45, 0.15, 0.30], *N* = 5
  *x_N_* = [0, 1.0, 0.45, 0.15, 0.30, 0.60, 0.75], *N* = 7
and **Case 2** with:
  *x_N_* = [0, 1.0, 0.15, 0.30, 0.45], *N* = 5
  *x_N_* = [0, 1.0, 0.15, 0.30, 0.45, 0.60, 0.75], *N* = 7

**Figure 3.**
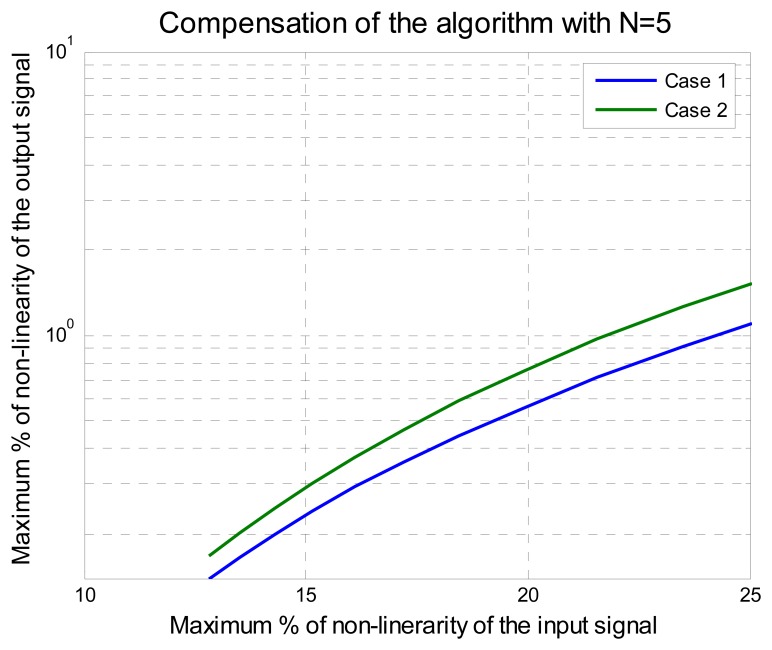
Algorithm response with five points of adjustment.

**Figure 4.**
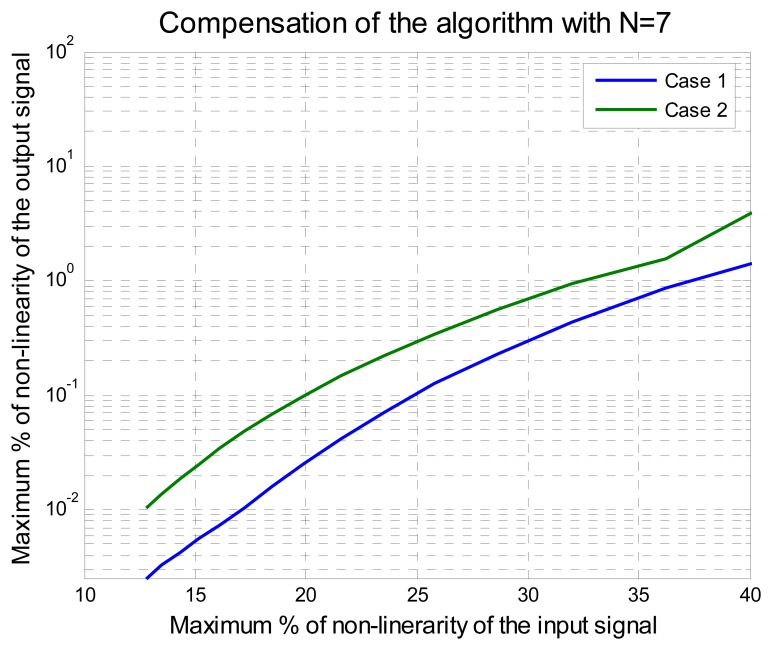
Algorithm response with seven points of adjustment.

In the experiment, the range of *x* was from 0 to 100 normalized from 0 to 1. The parameter *r* was changed from 95 to 1. The maximum percentage of nonlinearity relative error between Equation (14) and Equation (12), *ε_ry_*, was computed and the maximum percent of nonlinearity relative error *ε_ry_* goes from 12.96% to 94.33%. The maximum nonlinearity relative errors using *N*= *5* and *N*=*7*, are shown in Figure 3 and Figure 4, respectively.

According to these results, if five points of adjustment are used, then the method can compensate sensors with a maximum nonlinearity relative error under 21%, yielding a maximum nonlinearity relative error output lower than 1% as shown in Figure 3. It is important to notice the difference of results between Case 1 and Case 2. Notice that Case 2 has less error compared with the result of Case 1, the difference was the order of the elements *x*(3), *x*(4) and *x*(5) for *N*=5 that was taken.

According to these results, if seven points of adjustment are taken then the method can compensate sensors with a maximum nonlinearity relative error under 32%, yielding a maximum nonlinearity relative error output lower than 1% as shown in Figure 4. It is noteworthy that the difference of results between Case 1 and Case 2 with *N* = 7. Case 2 has lower error compared with the result of Case 1. The difference is related to the order of the elements, *x*(3) to *x*(7), supplied to compute the *f*_7_ function. The error difference between the results of Case 1 and Case 2 is important and it is noted in Case 2 of both experiments, using *N*=*5* and *N*=*7* adjustment points, the yields have lower error and the algorithm can compensate the sensor with the highest nonlinearity.

Another important observation from Figures 3 and 4 is that the nonlinearity relative error output changes not only with respect of the number of adjustment points used but also with the order of the adjustment points selected. In Figures 3 and 4, results showed that the error of Case 1 is greater than the error of Case 2. Therefore, this observation leads us to find a method to assure that the obtained result of the coefficients *k_N_* and the functions *f_N_* are optimal. This method is presented next.

### 3.2. Improved Polynomial Progressive Algorithm with Permutation Vector Analysis

In order to establish a systematic method to avoid all the subjective aspects of the original algorithm an improvement to the algorithm was performed. This is proposed herein through the analysis of the response of the polynomial progressive algorithm. Then, considering the adjustment input vector for *N*=*6*, lets say:

(15) $$x=[x_{1},x_{2},x_{3},x_{4},x_{5},x_{6}]$$

So a set of new vectors $x_{p}^{l}$ are generated and these correspond to the permutations of *x* but without permuting the vector elements *x*_1_ and *x*_2_, which represent the lower limit and the upper limit that can be measured by the measurement system or the intelligent sensor, where *p* denotes permutation and *l* denotes permutation number. For example if *x* is defined as:

(16) x=[0.0,1.0,0.15,0.30,0.45,0.60]

**Figure 5.**
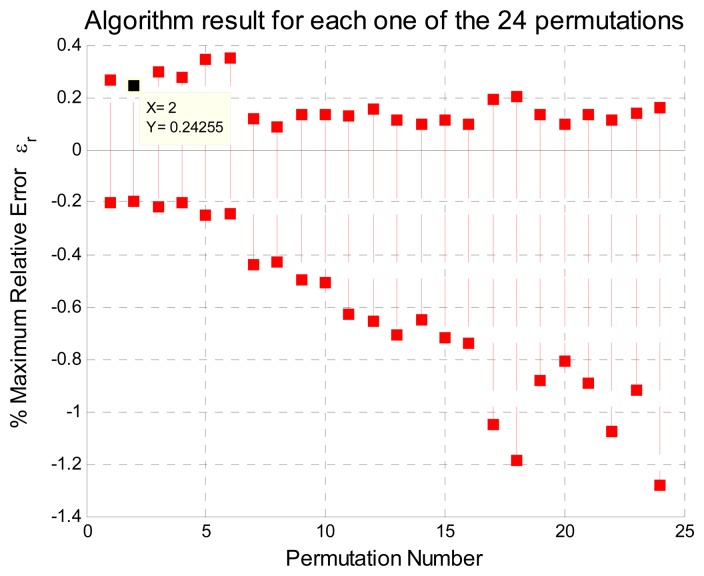
Percentage of nonlinearity error of PVA.

The numbers of $x_{p}^{l}$ vectors that can be generated in this case are 24 permuted vectors, that is, *l* goes from 1 to 24. The progressive polynomial method is then evaluated with each one of these permuted vectors. This process yields twenty-four $f_{N}^{l}$ functions; for this example *N* = 6 and *l* take values from 1 to 24. Figure 5 shows the nonlinearity relative error output for each *f*_6_ function. Considering an input signal with 28.87% of maximum nonlinearity relative error, the method will generate a maximum nonlinearity relative error output lower than 0.24%. This result is obtained with the permutation vector $x_{p}^{2}$, the number superscript 2 is to indicate the permutation number, defined by:

(17) $$x_{p}^{2}=[0.0,1.00,0.15,0.30,0.60,0.45]$$

As it can be noticed from Figure 5 the maximum nonlinearity relative error output using $x_{p}^{2}$ is lower or equal to the nonlinearity relative error output reported with *N*=*7*. This finding indicates that with the following proposed method, the permutation vector analysis (PVA), the order of the optimal *x_p_* can be found. Consequently, the empirical aspect to select the order of the adjustment points involved in the progressive polynomial method is avoided. To summarize, the PVA method provides a novel formal methodology to select the order of the adjustment points to assure the optimal compensation result and the minimum nonlinearity relative error. Programming this algorithm into the μC or DSP or central processing unit of the intelligent sensor; provides the sensor with the self-adjustment capability or the self compensation capability.

The method can be generalized as follows: with *N* adjustment points *x* = [*x*_1,_*x*_2,…,_*x_N_*], where *x*_1_ and *x*_2_ represent the lower and the upper limits that can be measured by the measurement system or intelligent sensor defining the (N–2)! PVAs:

(18) $$\begin{array}{cc} x_{p}^{l}=[x_{1},x_{2},x_{3},\ldots ,x_{N}], & l=1,2,\ldots \end{array},(N-2)!$$

The elements *x_1_* and *x_2_* are not permuted. The permutation number is denoted by *l*. Remember that for each $x_{p}^{l}$ exist its corresponding $y_{p}^{l}$ and $t_{p}^{l}$.

**Figure 6.**
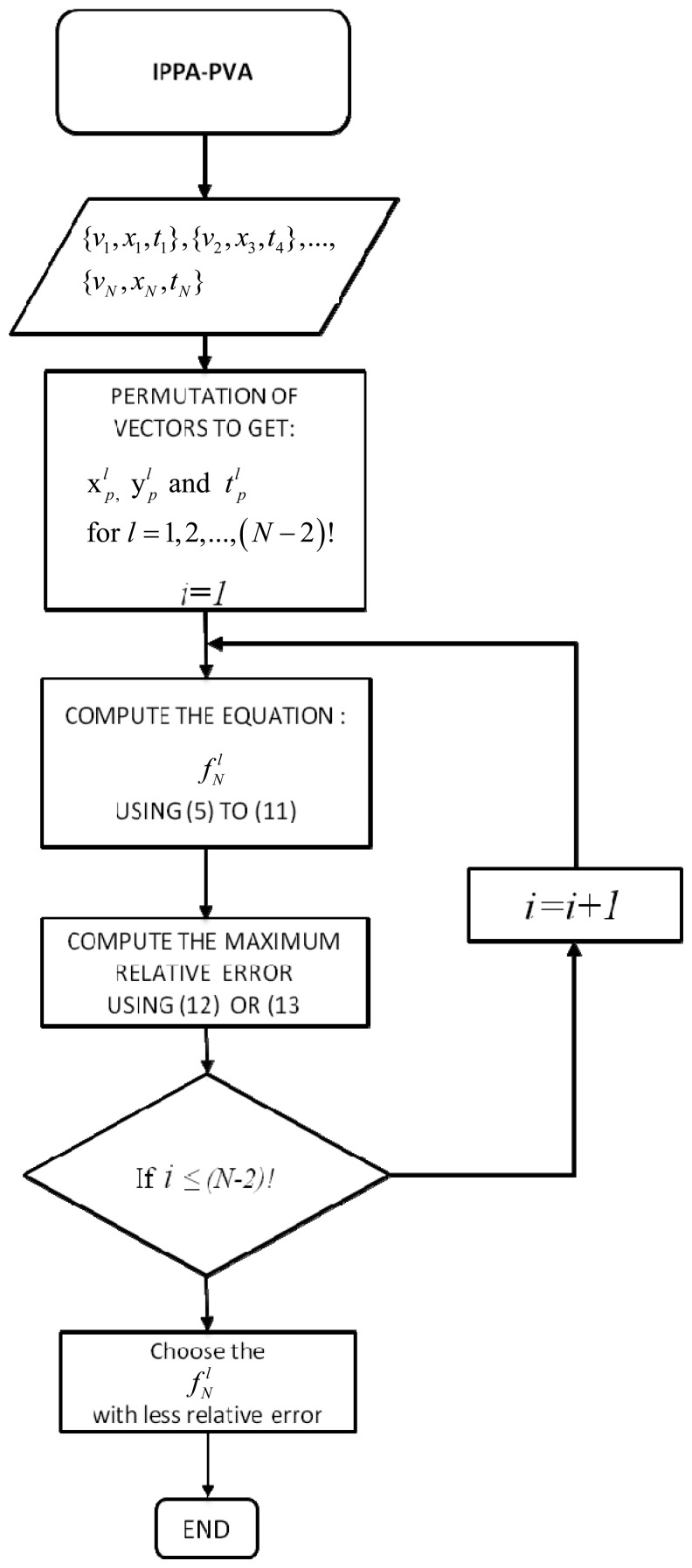
Flow chart of the PVA Algorithm.

Now, the $f_{N}^{l}$ functions are computed for each $x_{p}^{l}$, and the nonlinearity error associated using (12) is evaluated. The optimal solution that corresponds to the minimum error will be selected, this algorithm can be represented by the flow chart on Figure 6.

The following experiment was done using simulation software in order to evaluate the improved algorithm: First, five sets of readjustment points were taken; the difference between each set was the number of adjustment points *N*. Second, the input signal *x* for the algorithm was defined by (14) which is shown in Figure 2, the range of adjustment was 0 to 100. Third, the PVA algorithm described above was computed. The input signals *x* for the experiment after normalization process (2) and (3) were:

*x_N_* = [0, 0.15, 0.30, 0.45, 1], N=5
*x_N_* = [0, 0.15, 0.30, 0.45, 0.6, 1], N=6
*x_N_*= [0, 0.15, 0.30, 0.45, 0.60, 0.75, 1], N=7
*x_N_* = [0, 0.14, 0.28, 0.42, 0.56, 0.70, 0.84, 1], N=8
*x_N_* = [0, 0.12, 0.24, 0.36, 0.48, 0.60, 0.72, 0.84, 1], N=9

After the PVA, the new vectors $x_{p}^{l}$ with the right order to compute the $f_{N}^{l}$ function were found. This was done to confirm that the result was the optimal with a minimum percentage of nonlinearity. The new vectors $x_{p}^{l}$ were the result was optimal are:

$x_{p}^{2}=[0,1,0.15,0.45,0.30]$, N=5
$x_{p}^{2}=[0,1,0.15,0.60,0.45]$, N=6
$x_{p}^{6}=[0,1,0.15,0.30,0.75,0.60,0.45]$, N=7
$x_{p}^{6}=[0,1,0.14,0.28,0.42,0.84,0.70,0.56]$, N=8
$x_{p}^{108}=[0,0.12,0.24,1,0.12,0.24,0.84,0.48,0.72,0.60,0.36]$, N=9

**Figure 7.**
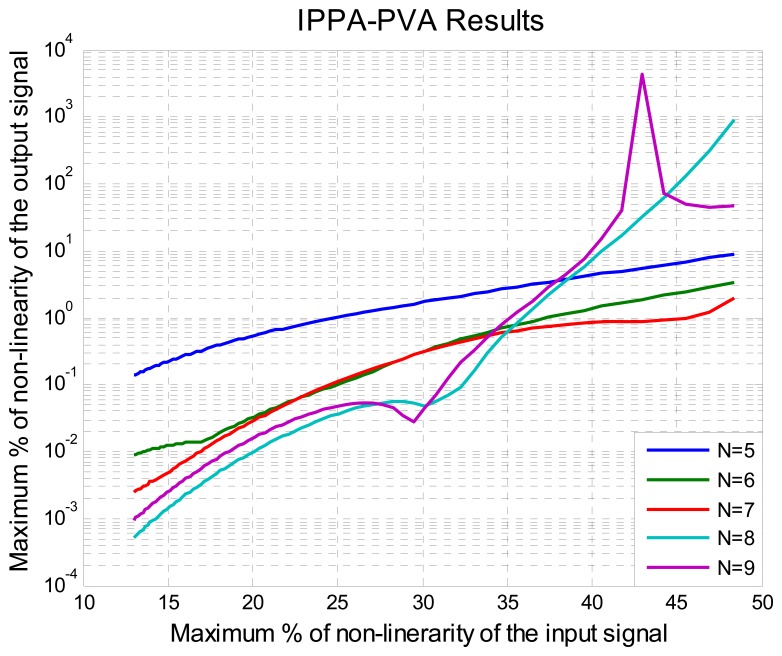
Results of the improved polinomial progressive algorithm for self-adjustment.

Keep in mind that $x_{p}^{108}$ indicates that the best result was found in the permutation 108 when the vector of adjustment was nine points. Then, the corresponding $f_{N}^{l}$ for the optimal function was computed and evaluated using the input signal of Figure 2, the results are shown in Figure 7.

By analyzing the results of Figure 7 we can see that with *N* = *5*, the improved polynomial progressive algorithm with permutation vector analysis (IPPA-PVA) can fix problems of nonlinearity up to 25% with less of 1% of relative error, instead of the 21.55 % obtained before the empirical application of the algorithm. Using *N* = 7 the improved algorithm and the kind of the input signal have the best results and can fix problems of nonlinearity up to 45% instead of 36% obtained before (see Figure 4). Besides, in order to get better results with the improved algorithm two important aspects needed to be considered here: the quantitative results of the algorithm capability and the improved method guarantee that the optimal result is always reached. With *N* = 8 and *N* = 9 the results were improved for input signal under 32% of nonlinearity but were not better for input signal upper to 32% of nonlinearity because oscillations were present around the solution, some author's call this the “over fitting” effect [35]. This is not a problem here because an algorithm that works with a small quantity of readjustment points was being sought. This in turn results in an improved algorithm and its practical application is presented in the next section.

## 4. Intelligent Sensor Design with IPPA-PVA for self-Adjustment implemented on small MCU

Temperature measurement systems are commonly used in almost any process. A thermistor as temperature sensor was selected in the construction of a measurement system. The thermistors, besides having a diversity of applications, can be found in a great variety of ways, sizes and characteristics. In this case, the major characteristic that will be analyzed is the *β* coefficient, fundamental characteristic to describe the nonlinearity error. For example values from *β*=*3100* to *β*=*4500* generate nonlinearity errors from 41.07% to 51.34%. With this percentage of error it is too difficult to obtain acceptable results with the described algorithm.

**Figure 8.**
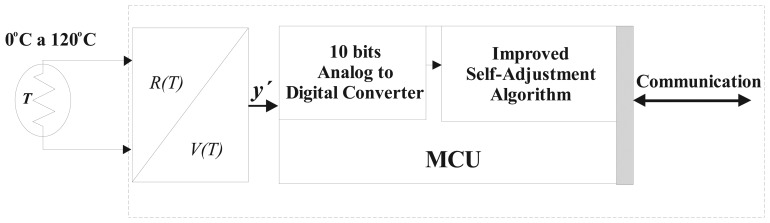
Topology of the intelligent sensor.

It is clear that in practical cases, the sensor will be on a circuit converting temperature to voltage capability, for example, a tension divider circuit or a Wheatstone bridge. The intelligent sensor was designed on a small MCU. Its topology is shown in Figure 8. The major features of the MCU for physic implementation are: eight bits word, ten bits analog to digital converter ADC, clock of 20 MHz, 3 Kbytes of RAM and communication of Control Area Network (CAN 2.0B). The improved algorithm described in Section 3 was programmed using C language.

**Figure 9.**
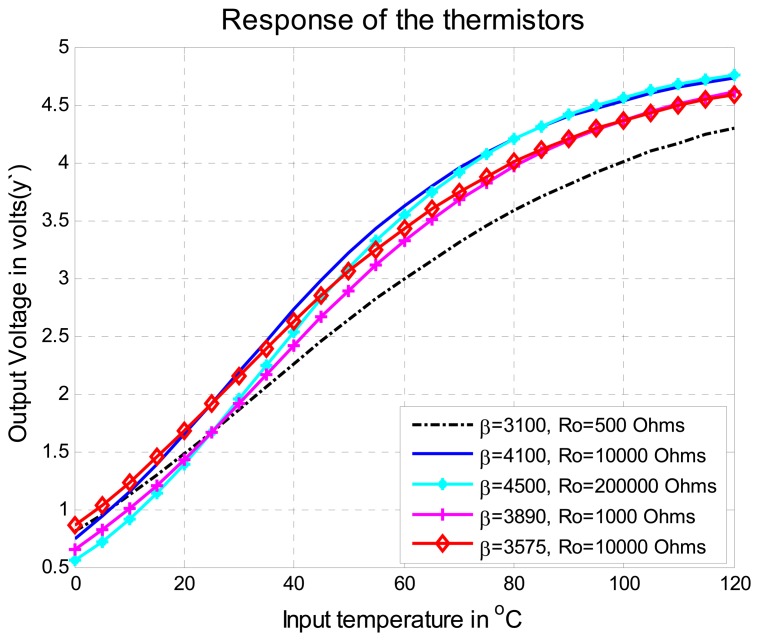
Response of thermistors used in the temperature measurement system (Ro is the resistance of the thermistor at 25°C).

**Figure 10.**
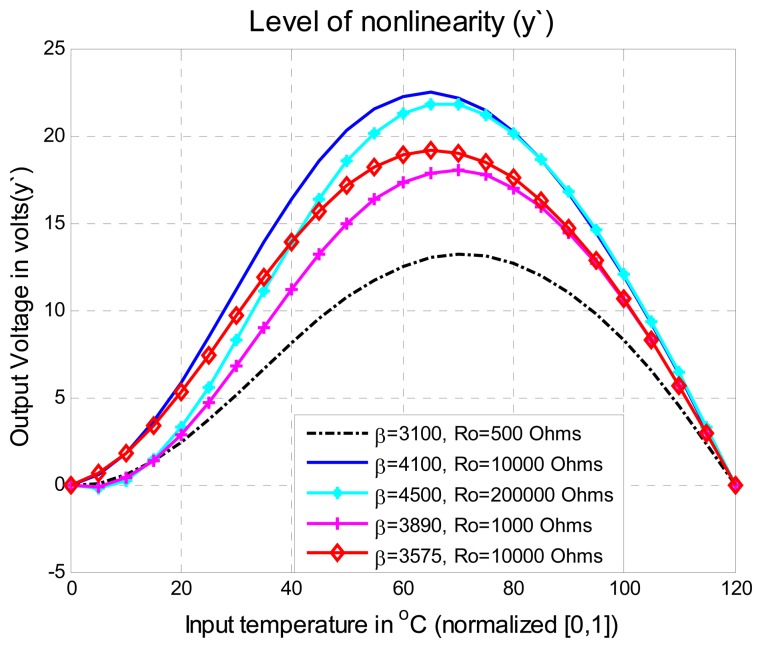
Feature of nonlinearity of the thermistors used in the temperature measurement system (Ro is the resistance of the thermistor at 25°C).

Five thermistors of different values of *β* in the range from 3,100 to 4,500 were selected and the range for the temperature measurement system was selected from 0°C to 120°C. In order to perform the test, each thermistor was placed on a tension divider circuit and the output was switched to the MCU. The output voltage of the tension divider circuit is the signal *y*′ of (1) shown in Figure 9.This signal presents offset problems and nonlinearity that will be fixed using the improved algorithm described above. The maximum percentage of nonlinearity of the output voltage was 13% with a thermistor of *β*=3890 and 22.7% with a thermistor of *β*=4100. On Figure 10, the nonlinearity feature of each thermistor is illustrated. Using the methodology presented in Section 3.2 and reviewing Figure 7, it can be noted that if six points are used to carry out the Self-Adjustment, the maximum percentage of nonlinearity of the output signal will be less than 1%.

The input signal *x*′ for the readjustment was a temperature of *x*′ = [0, 25,50,75,100,120] in °C. Using the Equation (2 and 3) and the thermistor with *β*=4100 the values normalized to the vectors **x**, **y** and **t** were calculated in order to obtain the vectors:

$$\begin{array}{c} x=[0,0.2083,0.4167,0.6250,0.8333,1]\;\text{y}=[0,0.2929,0.6251,0.8391,0.9530]\;\text{and}\\ t=[0,0.2083,0.4167,0.6250,0.8333,1] \end{array}$$

This signal was supplied to the improved algorithm. The results are presented in the next section.

## 5. Tests and Results

**Figure 11.**
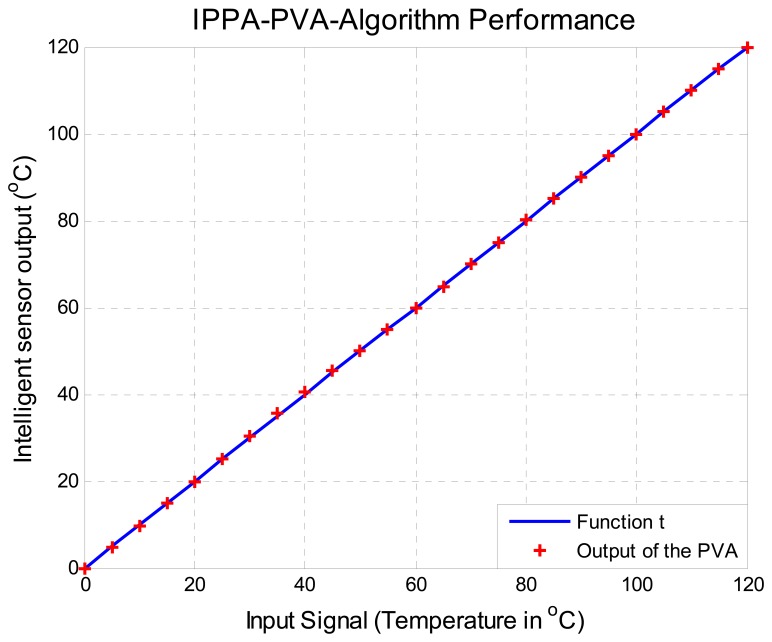
Improved algorithm performance with six readjustment points

The proposed improved algorithm was computed and the performance of the improved algorithm was compared against a Honeywell temperature meter, number UDC3000 with a thermocouple type K, span of −29 to 538°C (−20 to 1,000°F) and accuracy of ± 0.02%, taking 25 measures from a range of 0 to 120°C in 5°C steps using an oven system to change the temperature. The results are shown in Figure 11.

**Figure 12.**
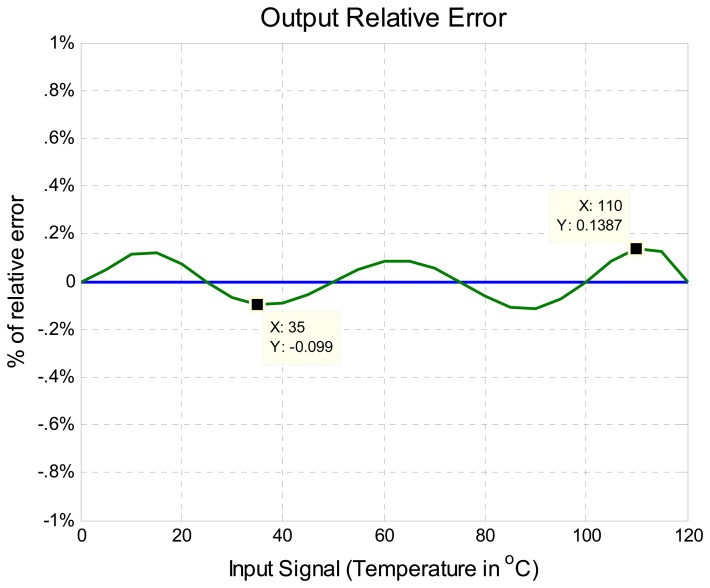
Improved algorithm relative error with six readjustment points

The output of the improved algorithm compared with the target straight line can be seen in Figure 11. In order to better visualize the error between the algorithm output and the target straight line the percentage of relative error of nonlinearity computed with Equation (12) is shown in Figure 12. In summary Figure 12 shows the difference between the ideal output and the output provided by the Improved Algorithm. It can also be observed that the maximum percentage of nonlinearity relative error of is under 1%, approximately 0.13% bellow of 1% as was estimated from simulation and was illustrated in Figure 7 with six calibration points.

The same process as described above was done for the other thermistors and the obtained results are shown in Table 1. The thermistor characteristics are listed on the first column. Column two shows the percentage of nonlinearity of the output voltage. The best permutation of the vector $x_{p}^{l}$ and its maximum percentage of nonlinearity error range obtained are shown in Columns 3 and 4, respectively.

The results indicate that the maximum nonlinearity relative error, for each case, is less than 1%. These results were generated from a quantitative analysis of IPPA-PVA, therefore, yielding the certainty of confidence that it is the optimal solution.

Moreover, it is important to comment on the sensor resolution. The ADC determinates the sensor resolution and this is defined by: $\text{Resolution}=\overset{E_{\text{FSR}}}{\;}/\underset{2^{n-1}}{\;}$, were *E_FRS_* is the full range voltage scale and the number of bits of the ADC is *n*. In our example the *E_FRS_* = 5 volts and the ADC is ten bits, then the sensor resolution is 9.8 *mV*, meaning that the temperature sensor is limited to detect temperature changes of about 0.3 °C. If this resolution represents a problem in a specific application, an external ADC with more than 10 bits can be used.

## 6. Conclusions

In this paper an improved progressive polynomial algorithm to perform compensation in intelligent sensors was presented. The improved algorithm needs to be executed only the first time the sensor is calibrated. Using data from Figure 7, which was discussed early, the position of the calibration points changes according to the percentage of nonlinearity of the sensor output signal. Then the initial value of the percentage of nonlinearity can be saved as an important sensor specification data and the process will be repeated only if a substantial change in this value is presented. Beside a special methodology to evaluate the capability of any compensation algorithm with one quantitative parameter was presented. In this way, any doubt can be avoided and a significant amount of time can be saved for the designers.

The proposed method assures the optimal solution using few adjustment points, reducing the time spent in the adjustment of the calibration process and reducing the calibration cost as a consequence. This method can be used for any sensor, as long as, the percentage of nonlinearity is under 40% and if the desired error is less than 1%.

The algorithm can be easily programmed into a microcontroller because it consists of simple operations and only the coefficients from *k*_1_ to *k_N_*, needed to be saved.

## Figures and Tables

**Table 1.** Improved Algorithm evaluation results.

Thermistors Features	% max. initial error ε_r_	Order of the $x_{p}^{l}$ vector	% max ε_r6_ of $f_{6}^{l}$

*β*=3100; To=25°C Ro=500 Ohms	13.20%	$x_{p}^{14}=[0,120,75,25,100,50]$	-0.043 a 0.0192
*β*=4100; To=25°C Ro=10000 Ohms	22.47%	$x_{p}^{2}=[0,120,25,50,100,75]$	-0.099 a 0.1383
*β* =4500; To=25 Ro=200000 Ohms	21.79%	$x_{p}^{2}=[0,120,25,50,100,75]$	-0.155 a 0.1931
*β* =3890; To=25°C Ro=1000 Ohms	18.02%	$x_{p}^{4}=[0,120,25,75,100,50]$	-0.099 a 0.091
*β* =3575; To=25°C Ro=10000 Ohms	19.17%	$x_{p}^{2}=[0,120,25,50,100,75]$	-0.0493 a 0.0718
